# Undifferentiated connective tissue disease presenting with prevalent interstitial lung disease: Case report and review of literature

**DOI:** 10.1186/1746-1596-6-50

**Published:** 2011-06-07

**Authors:** Francesca Lunardi, Elisabetta Balestro, Beatrice Nordio, Franco Cozzi, Roberta Polverosi, Paolo Sfriso, Fausto Braccioni, Fiorella Calabrese

**Affiliations:** 1 Department of Diagnostic Medical Sciences and Special Therapies, University of Padua, Padua, Italy; 2 Department of Cardiac, Thoracic, and Vascular Sciences, University of Padua, Padua, Italy; 3 Department of Clinical and Experimental Medicine, University of Padua, Padua, Italy

## Abstract

Undifferentiated connective tissue diseases (UCTDs) are clinical entities characterised by signs and symptoms suggestive of a systemic autoimmune disease, which do not fulfil the diagnostic criteria for a defined connective tissue disease. Lung involvement can complicate the course and management of the disease, often determining a worse outcome. Respiratory dysfunction as the first clinical manifestation has seldom been reported.

We describe a case of a female patient who developed significant respiratory dysfunction as the principal clinical sign. Video-assisted thoracoscopy was performed and a histological pattern of nonspecific interstitial pneumonia (NSIP) was found. A pathological diagnosis suggested careful follow-up with extensive immunological screening which then detected Raynaud's phenomenon and positivity of antinuclear antibodies. After a multidisciplinary discussion (pneumologist, radiologist, pathologist and rheumatologist) a final diagnosis of NSIP associated with UCTD was made. The diagnosis of UCTD should be considered when NSIP is diagnosed even in cases with evident first clinical manifestations of severe respiratory dysfunction. A multidisciplinary approach in the field of interstitial lung disease with NSIP, also including rheumatologic expertise, is fundamental to achieve a prompt and correct diagnosis.

## Background

The term undifferentiated connective tissue disease (UCTD) is used to define clinical entities characterised by features suggestive of CTD which do not meet the classification criteria of the American College of Rheumatology for a specific single disease, such as systemic lupus erythematosus, systemic sclerosis, polymyositis/dermatomyositis, and Sjögren's syndrome [1-9]. UCTD can evolve in these patients over time.

As only a few reports have described lung involvement during UCTD the natural history still remains unknown and unpredictable. Interstitial lung disease (ILD) with a histological pattern of non specific interstitial pneumonia (NSIP) has recently been reported to be the most frequent lung manifestation [10-13], usually responsible for progression and adverse outcome of the disease. Lung involvement as the first clinical manifestation of UCTD is rarely reported. We discuss the case and review the associated literature of UCTD.

## Case Presentation

### Clinical summary

A 50-year-old non-smoking female textile worker after a flu-like episode started to suffer from dry cough and progressive dyspnoea. Chest radiography showed parenchymal pulmonary opacity in the lower posterior segment of the right haemithorax and bilateral interstitial thickening of the lower pulmonary segment. The patient was therefore treated with antibiotics and steroids but the clinical and radiological manifestations did not improve.

**Figure 1:**
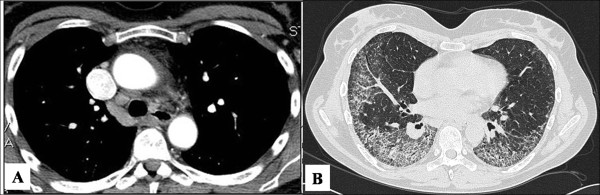
**Computed tomography scan of the lung**. A: Axial CT scan shows right paratracheal linfoadenopathy B: Axial HRCT scan shows septal interlobular thickening with ground glass opacities at the lower lobes

Computed tomography (CT) scan of the lungs three months later revealed a ground glass pattern in the apical segment of the upper and lower lobes. Lymphadenopathy in the paratracheal space was also observed (Figure 1). The laboratory results showed an increased level of inflammatory parameters (erythrocyte sedimentation rate: 78 mm; C-reactive protein: 5.7 mg/dl; Fibrinogen: 492 mg/dl), without any other positive tests (e.g. rheumatoid factor). The pulmonary function test showed a restrictive pattern (vital capacity: 1.59 L, 50% predicted; total lung capacity: 3.94 L, 75% predicted) with a reduction in the single breath diffusion capacity of carbon monoxide (TLCO/VA 0.84 mmol/min/kPa/l, 52% predicted). Arterial oxygen tension at rest was normal (pH: 7.44; pO_2_: 92.4 mmHg; pCO_2_: 38.8 mmHg). A six-minute walking test revealed oxygen desaturation (87%) with severe dyspnoea (BORG scale 5/10). Bronchoscopy with combined bronchoalveolar lavage and transbronchial biopsies were performed, but they provided no significant additional information.

Open lung biopsy was performed and based on a histological diagnosis of non specific interstitial pneumonia (NSIP) an accurate immunological follow-up was required.

**Figure 2:**
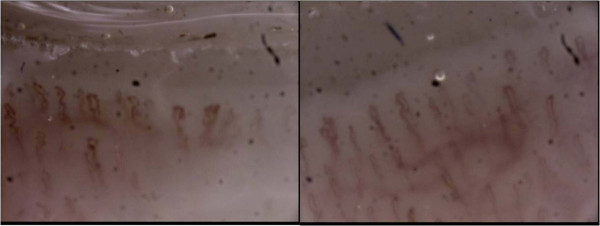
**Capillaroscopy images**. Abnormal capillary shape with winding and acrocyanotic capillaries.

The assay of a larger panel of autoantibodies showed only positivity of antinuclear antibodies (ANA), at titer 1/160. The other investigated antibodies (such as anti-ENA, anti-DNAds, anti-centromere, anti-cyclic citrullinated peptide and anti-tRNA synthetases) were all negative. Finally, a nailfold capillaroscopy (NFC) was recommended and showed the presence of minor capillary changes characteristic of a nonspecific pattern (Figure 2). Clear-cut signs of Raynaud's phenomenon (RP) were then reported during rheumatologic follow-up. After a multidisciplinary discussion (pneumologist, radiologist, pathologist and rheumatologist) a final diagnosis of NSIP associated with UCTD was made.

Therefore, the patient was discharged and started a treatment regimen of oral cyclophosphamide (100 mg/die) and prednisone (25 mg/die) for 12 months. The combination was then stopped and the patient was followed only with low dose of corticosteroids.

Up to now, over a follow-up period of three years, the clinical picture and the pulmonary function test remain stable and no definitive CTD has developed.

### Pathological findings

**Figure 3:**
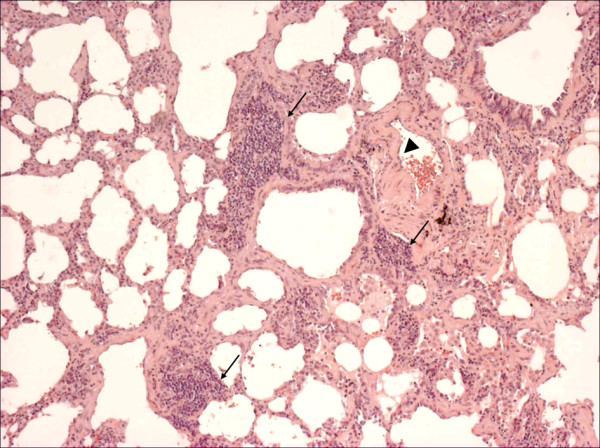
**Histological pattern in lung biopsy**. Panoramic view showed interstitial inflammatory cell infiltration sometimes aggregated in follicular pattern (arrows). Vessel remodelling (arrowhead) is also well seen (original magnification, haematoxylin-eosin stain: 40x).

Low-magnification microscopy showed an uniform alveolar septa thickening by cellular infiltrate and fibrosis. At higher magnification, the alveolar septal interstitium was expanded by mild inflammation and collagen deposition with minimal honeycomb changes. No fibroblastic foci and active fibrosis were seen and patchy lymphoid aggregates were occasionally visible. The interstitial chronic inflammation consisted of lymphocytes and some plasma cells. Increased macrophagic infiltration was seen in some alveolar spaces. The appearance was consistent with the fibrosing variant of NSIP (Figure 3). Organising pleuritis with mesothelial hyperplasia was present. Medial vessel thickening was particularly seen in remodelled lung areas.

## Conclusions

Up to 25% of patients who show clinical and laboratory features of systemic autoimmune diseases do not fulfil the classification criteria of ACR for a specific CTD and they are considered to have a distinct clinical entity, called UCTD [1,4,7,8]. The definition is still under debate, although it is becoming more definite. In 1999 and 2006 Mosca *et al. *reviewed the literature and proposed that preliminary classification criteria for UCTD include the following: at least one clinical manifestation of CTD, positive ANA results, and a disease duration of at least three years [4,8].

It may be difficult to distinguish these conditions from early phases of defined diseases such as systemic lupus erythematosus, systemic sclerosis and others. The natural history of these rare entities is variable: a high percentage of patients with UCTD maintain an undifferentiated clinical course and do not evolve to a distinct CTD, whereas some patients can evolve over time [4,7,8,11].

Laboratory test screening is essential to identify markers that may suggest a systemic, autoimmune disease, or specific organ involvement. In fact, about 90% of the patients with UCTD show positive ANA [1,5]. Capillaroscopy is useful to detect abnormalities in capillary shape and blood flow, which suggest features of Raynaud's phenomenon.

The onset of UCTD is similar to most CTDs, peaking in the middle years of life. There are no specific signs or symptoms of UCTDs because these entities present manifestations common to other CTDs. The main clinical features at onset of UCTD are arthritis and arthralgia, Raynaud's phenomenon, pleuritis and pericarditis, xerostomia and xerophtalmia, leukopenia, esophageal involvement, fever and myositis [1,9,14]. Major organ involvement is unusual and the lung has been reported as a late complication, often determining a worse outcome [15].

Recently an ATS working group described idiopathic NSIP as a distinct entity that occurs mostly in middle-aged women who never smoked and who often have positive serologic tests (antinuclear antibodies or rheumatoid factor) [16].

The typical histological findings of NSIP detected in our patient who also presented the clinical phenotype (middle-aged, non-smoking woman) suggested an additional careful investigation for an accurate rheumatologic evaluation and follow-up.

Kinder *et al. *recently showed that the majority of patients (88%) who met the UCTD criteria had distinct radiological and pathological features of NSIP, thus emphasizing that this pattern is peculiar of the disease [10]. The same group more recently highlighted the concept that patients with UCTD and lung involvement have improved pulmonary function during the follow-up, as in our case [11] compared to other unfavourable interstitial diseases as idiopathic pulmonary fibrosis.

Lung involvement usually appears as a complication in established UCTD. There is only a recent study which has demonstrated that NSIP may be the first clinical manifestation of the disease [13], as in our case. The prospective study by Romagnoli *et al. *demonstrated that more than 50% of patients diagnosed with NSIP showed an autoimmune disease, as UCTD, in their follow-up. In particular, UCTD was detected in 22% of NSIP patients [13].

In conclusion, we have described a case of a patient with UCTD whose first clinical manifestation was lung involvement. UCTD should be considered when NSIP is diagnosed even in cases with evident first clinical manifestations of severe respiratory dysfunction.

A multidisciplinary approach with the intervention of pneumologists, radiologists, pathologists and rheumatologists is recommended to achieve a correct diagnosis.

## Consent

Written informed consent was obtained from the patient for publication of this case report and any accompanying images. A copy of the written consent is available for review by the Editor-in-Chief of this journal.

## Competing interests

The authors declare that they have no competing interests.

## Authors' contributions

BN and EB were involved in the clinical assessment of the patient and in paper writing, FL in morphological description and paper writing, FC and PS in rheumatological investigations, FB in clinical assessment, RP in radiological evaluations, FC in pathological diagnosis and paper writing.

All authors read and approved the final manuscript.
